# Biomimetic Action of Zinc Hydroxyapatite on Remineralization of Enamel and Dentin: A Review

**DOI:** 10.3390/biomimetics8010071

**Published:** 2023-02-08

**Authors:** Andrea Butera, Carolina Maiorani, Simone Gallo, Maurizio Pascadopoli, Martina Quintini, Marco Lelli, Fabrizio Tarterini, Ismaela Foltran, Andrea Scribante

**Affiliations:** 1 Unit of Dental Hygiene, Section of Dentistry, Department of Clinical, Surgical, Diagnostic and Pediatric Sciences, University of Pavia, 27100 Pavia, Italy; 2 Unit of Orthodontics and Pediatric Dentistry, Section of Dentistry, Department of Clinical, Surgical, Diagnostic and Pediatric Sciences, University of Pavia, 27100 Pavia, Italy; 3 Department of Industrial Chemistry “Toso Montanari”, University of Bologna, 40126 Bologna, Italy; 4 Incos-Cosmeceutica Industriale, Funo di Argelato, 40050 Bologna, Italy

**Keywords:** biomimetic, zinc-hydroxyapatite, enamel, dentin, remineralization, dentistry

## Abstract

Biomimetic zinc–carbonate hydroxyapatite technology was developed to realize materials that mimic the natural hydroxyapatite of enamel and dentin and possess good activity in terms of affinity to adhere to these biological tissues. The chemical and physical characteristics of this active ingredient allows the hydroxyapatite itself to be particularly similar to dental hydroxyapatite, enhancing the bond between biomimetic hydroxyapatite and dental hydroxyapatite. The aim of this review is to assess the efficacy of this technology in terms of benefits for enamel and dentin and reduction of dental hypersensitivity. Materials and methods: A literature search (Pubmed/MEDLINE and Scopus) of articles from 2003 to 2023 was conducted to analyze studies focused on the use of zinc-hydroxyapatite products. Duplicates were eliminated from the 5065 articles found, leaving 2076 articles. Of these, 30 articles were analyzed based on the use of products with zinc–carbonate hydroxyapatite in these studies. Results: 30 articles were included. Most of the studies showed benefits in terms of remineralization and prevention of enamel demineralization in terms of occlusion of the dentinal tubules and reduction of dentinal hypersensitivity. Conclusion: Oral care products such as toothpaste and mouthwash with biomimetic zinc–carbonate hydroxyapatite were shown to provide benefits according to the aims of this review.

## 1. Introduction

The contribution of good personal oral hygiene practice for maintaining healthy teeth and gums has long been recognized. The practice of using a toothbrush together with a paste or powder to augment the mechanical cleaning action has found universal acceptance [1,2,3].

The introduction of toothpaste with additional active ingredients, such as fluoride, anti-bacterial agents and remineralization systems has further enhanced the beneficial effect derived from tooth cleaning [4,5,6].

In particular, the widespread use of fluoride toothpaste to combat dental caries has led to a global reduction of disease [7]. In the first instance, research on the anti-caries efficacy of fluoride was focused on its ability to reduce enamel demineralization caused by plaque acids [8]. In the course of extensive work on the underlying mode of action, it became clear that fluoride is not only effective in reducing enamel demineralization but also in enhancing enamel remineralization [9].

These findings firmly established our current understanding of the onset and progress of dental caries as a dynamic process. Firstly, the enamel is subjected to demineralization or remineralization depending on the frequency and duration of high or low pH conditions at the tooth surface [10,11]. There are factors that accelerate the process of demineralization of teeth, such as high intake of acidic foods (fruit, citrus, Coca-Cola, wine and soda), eating often and not brushing teeth regularly after meals. Stagnation of food in the mouth increases the action of bacteria that attack the enamel of the teeth, as does contact with acidic substances of gastric juices (gastroesophageal reflux and vomiting) [12]. At an important second level, the erosion of teeth and the demineralization/remineralization balance will be determined by the availability at the tooth surface of calcium and phosphate ions, or ions capable of modifying the demineralization/remineralization process, such as fluoride [13,14]. It was also found that dental erosion is governed by the same physical and chemical processes and conditions, because they can represent the initial stage of possible future caries [15].

The natural source of calcium and phosphate is saliva, and research has shown that high saliva calcium levels are strongly correlated with low caries [16,17]. In contrast, agents like fluoride have to be provided from external sources, such as mouthwash, toothpastes or gums [18].

It has also been realized that an external supply of calcium or calcium-phosphate minerals could be delivered with toothpaste to be deposited directly on the enamel surface, thus providing an immediate remineralization effect and also generating high calcium and phosphate concentrations at the tooth surface [18,19]. This led to the development of toothpaste ingredients that could fulfil this role.

Various products have been placed on the market whose active principle is represented by a substituted zinc–carbonate hydroxyapatite. It is possible to define this particular hydroxyapatite as biomimetic for two reasons. The first is related to the substitution present within the hydroxyapatite crystals themselves (this makes each hydroxyapatite biomimetic by definition). The second is linked to the fact that this particular hydroxyapatite is identical to the target, and therefore to the hydroxyapatite of the tooth, thanks to its chemical and physical characteristics (shape, size, ionic substitution). Reviews have given insight into the wide range of research in this field and discussed its application in oral care products [20,21].

It is therefore evident that in order to have a product with good adhesion properties to the dental surface, and which is therefore able to remineralize (deposit a new mineral phase on) the tooth, it is necessary to have an active ingredient that has a high affinity and similarity with the hydroxyapatite of the tooth. It is evident that to achieve such a result, it is not sufficient to use a commercial hydroxyapatite [22]. To obtain this result, a particular technology has been developed in which microcrystals of biomimetic hydroxyapatite are created or replaced with the main substituents present in our teeth (zinc ion and carbonate ion). This process, together with its chemical and physical characteristics (shape, size, surface area, degree of crystallinity) makes this hydroxyapatite particularly reactive on the surface and therefore able to bind to the dental surface. In fact, only zinc–carbonate hydroxyapatite is particularly reactive on the dental surface with both enamel and dentin.

The reason for this depends on the particular biomimicry of zinc–carbonate hydroxyapatite, which is similar in ionic substitution, morphology and size to that of enamel and dentin. Although there are currently few randomized clinical studies on the use of zinc–carbonate hydroxyapatite, there are promising results on its ability to remineralize enamel lesions and prevent or reduce demineralization [23,24,25,26].

Other enamel and dentine remineralization systems are widely used in dentistry, including fluorine and amorphous calcium phosphate-phosphopeptide casein.

Fluoride acts against demineralization because it is able to reduce the solubility of dentine and enamel. It has been shown to induce the formation of fluoroapatite (FA) or fluorinated hydroxyapatite (FHA), which are less soluble crystals than stoichiometric hydroxyapatite (HA). When brushing with a toothpaste containing fluoride, this is released and saved in different parts of the oral cavity such as soft tissues, dental surfaces, materials and prosthetic devices, and also in the context of bacterial biofilm. With repeated use over time, fluoride can become part of the crystalline structure of the hydroxyapatite, taking the place of the hydroxyl ion and forming fluoroapatite in the post-eruptive phase of the dental element. Toothpastes with a high fluorine content (>1500 ppm) have proved useful in increasing the level of fluoride in saliva throughout the day, changing the acidogenicity of interproximate plaque, increasing the buffering activity of saliva and decreasing the level of *Streptococcus mutans* [27,28,29].

Casein phosphopeptides derived from enzymatic hydrolysis are known as casein phosphopeptides (CPP). These bind calcium and phosphate ions through their multiple phosphor residues in an amorphous and bioavailable form. The resulting complexes are known as amorphous calcium phosphopeptide-phosphate casein (CPP-ACP). Complexes incorporating fluoride are known as calcium amorphous phosphate casein phosphopeptide-fluoride (CPP-ACPF). It is known that CPP-ACP complexes inhibit enamel demineralization and promote remineralization of early lesions of the enamel surface layer [30,31]. However, they are less effective than traditional fluoride toothpastes [32].

## 2. Materials and Methods

The present review aims to analyze the scientific literature relating to this technology (biomimetic zinc–carbonate hydroxyapatite) as applied in oral care products. The focus of the review is on linking this technology (through trials that have been conducted and published) to oral health benefits.

### 2.1. Eligibility Criteria

The following inclusion criteria were applied:

1. Type of Study. Case-control, cross-sectional, cohort studies, clinical trials, in vitro studies, reviews and meta-analyses from 2003 to 2023.
2. Type of Participant. Participants who used zinc–carbonate hydroxyapatite toothpaste and/or mouthwash.
3. Type of Intervention. Case-control, cross-sectional, cohort studies and clinical trials that have evaluated the possible benefits of zinc–carbonate hydroxyapatite toothpaste and/or mouthwash.
4. Outcome Type. Each variable included in the studies was taken into account.

### 2.2. Search Strategy

The review is based on the examination of studies identified through bibliographic research in the electronic databases Pubmed/MEDLINE and Scopus and by examining the bibliographies of the articles. Initially, all study abstracts were taken into consideration.

### 2.3. Research

We performed the search using the following keywords: “zinc hydroxyapatite” AND “enamel”, “zinc hydroxyapatite” AND “dentin”, “hydroxyapatite” AND “enamel”, “hydroxyapatite” AND “dentin”, “apatite” AND “enamel”, “apatite” AND “dentin”, “biomimetic” AND “enamel”, and “biomimetic” AND “dentin”, with a temporal range from 2003 to 2023.

### 2.4. Screening and Selection of Articles

**Figure 1:**
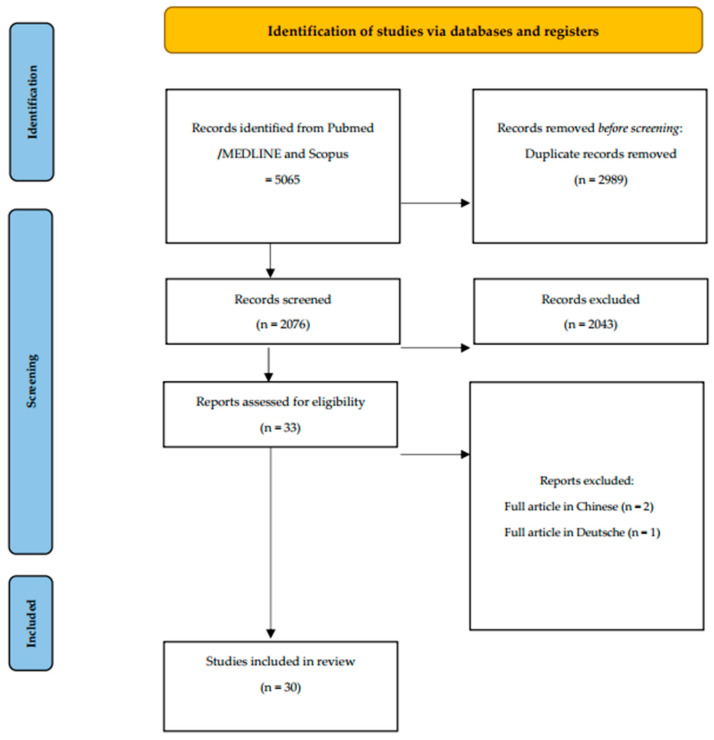
Flow chart of included studies: from 5065 articles, duplicates were eliminated, leaving 2076 articles; of these, 30 articles were analyzed based on use of products with zinc hydroxyapatite.

The search produced 5065 titles matching the search keyword. The following flowchart shows the selection criteria used to select the final 30 articles used for the review analysis (Figure 1). The results were filtered for relevance to toothpaste and mouthwash with zinc hydroxyapatite. The search yielded 30 published papers.

Articles were analyzed and grouped to assess the benefits provided in the prevention of tooth decay, action against dental erosion and effectiveness in combatting tooth hypersensitivity.

## 3. Results

The studies were divided based on evidence to assume the effectiveness of the tested products in terms of enamel/dentin protection (tooth decay and/or dental erosion) and reduction of dental hypersensitivity.

### 3.1. Enamel Protection (Tooth Decay and/or Erosion) [33,34,35,36,37,38,39,40,41,42,43,44,45,46,47,48,49,50,51,52,53], Table 1

Methods: The 21 studies selected for the review were published in English. Most were in vitro studies, conducted mainly in Italy and Germany (62% and 19%, respectively). Other studies were conducted in Switzerland, India, Egypt and the Czech Republic.

Participants: Most of the studies were conducted in vitro, using extracted human teeth or bovine teeth.

Intervention: The effects of toothpastes (85.7%) and mouthwashes (9.5%) with zinc hydroxyapatite were evaluated in the studies; one study evaluated the combined effect of toothpaste and mouthwash.

Outcomes: The use of the tested products has shown effectiveness in terms of remineralization and prevention of enamel/dentin demineralization, and in terms of protection against dental erosion processes (Table 1).

**Table 1:** Studies focused on erosion and dental caries.

Articles	Study	Agents	Conclusion
Bossù et al., 2019 [33]	In vitro/clinical trial	Zinc–carbonate hydroxyapatite toothpaste (undeclared percentage) ZnCO_3_/n-HAp	The use of Biomimetic hydroxyapatite toothpastes has proven to be a valuable prevention measure against dental caries.
Poggio et al., 2010 [34]	In vitro	Zinc–carbonate hydroxyapatite toothpaste (20%) ZnCO_3_/n-HAp	The toothpastes tested (Pronamel and BioRepair Plus) offer a degree of protection from erosive drinks.
Poggio et al., 2014 [35]	In vitro	Zinc–carbonate hydroxyapatite toothpaste (20%) ZnCO_3_/n-Hap Zinc–carbonate hydroxyapatite toothpaste (24%) ZnCO_3_/n-HAp	Biorepair Plus-Total Protection^®^ and Sensodyne Repair & Protect^®^ provided higher protective effect against dentin demineralization.
Colombo et al., 2017 [36]	In vitro	Zinc–carbonate hydroxyapatite toothpaste (undeclared percentage) ZnCO_3_/n-HAp	In this study treatment of erosively challenged enamel with Zn-Hap toothpaste showed a clear protective effect.
Lombardini et al., 2014 [37]	In vitro	Zinc–carbonate hydroxyapatite toothpaste (20%) ZnCO_3_/n-HAp Zinc–carbonate hydroxyapatite toothpaste (24%) ZnCO_3_/n-HAp	BioRepair Plus-Sensitive Teeth, Biorepair Plus-Total Protection and Sensodyne Repair & Protect provided lower effectiveness in protecting enamel against erosion.
Colombo et al., 2017 [38]	In vitro	Zinc–carbonate hydroxyapatite toothpaste (undeclared percentage) ZnCO_3_/n-HAp	The results of this study confirmed the potential benefit the Zn-HAP technology could provide in protecting enamel from erosive acid challenges. The treatment of erosively challenged enamel with Zn-Hap toothpaste showed a clear protective effect.
Bradna et al., 2016 [39]	In vitro	Zinc–carbonate hydroxyapatite toothpaste (24%) ZnCO_3_/n-HAp	Results revealed that toothpastes with strong potential to form acid-resistant deposits on the enamel surface and having low abrasivity should be used for effective prevention of enamel erosion.
Aykut-Yetkiner et al., 2014 [40]	In vitro	Zinc–carbonate hydroxyapatite toothpaste (undeclared percentage) ZnCO_3_/n-Hap	Toothpastes with anti-erosive formulations reduced dentine erosion, especially under simulated extrinsic erosive conditions, but were not superior to a conventional fluoride toothpaste.
Ganss et al., 2011 [41]	In vitro	Zinc–carbonate hydroxyapatite toothpaste (undeclared percentage) ZnCO_3_/n-Hap	Conventional NaF toothpastes reduced erosive tissue loss, but had limited efficacy regarding the prevention of brushing abrasion. The special formulations were not superior or were even less effective.
Chandru et al., 2020 [42]	In vitro	Zinc–carbonate hydroxyapatite toothpaste (undeclared percentage) ZnCO_3_/n-HAp	As per statistical analysis, maximum remineralization of enamel blocks occurred after applying Colgate Sensitive Plus^®^ toothpaste followed by BioRepair^®^ tooth paste and Regenerate enamel Science™ toothpaste. The least remineralization potential was shown by control group.
Kensche et al., 2016 [43]	In vitro/clinical trial	Zinc–carbonate hydroxyapatite toothpaste (undeclared percentage) ZnCO_3_/n-HAp	The biomimetic materials reduced ion release, but their effect was less pronounced.
Kranz et al., 2022 [44]	In vitro	Zinc–carbonate hydroxyapatite toothpaste (undeclared percentage) ZnCO_3_/n-HAp	Treatment with Biorepair^®^ did not affect enamel surfaces as proposed. Minor mineral precipitation and a reduction in surface roughness were detected on dentin surfaces only.
Alessandri Bonetti et al., 2014 [45]	In vitro	Zinc–carbonate hydroxyapatite toothpaste (20%) ZnCO_3_/n-HAp	The lowest grade of damage was recorded in samples brushed with Zn-CHA.
Tschoppe et al., 2011 [46]	In vitro	Zinc–carbonate hydroxyapatite toothpaste (24%) ZnCO_3_/n-HAp	With the in vitro conditions chosen, toothpastes containing n-HAp revealed higher remineralizing effects compared to amine fluoride toothpastes with bovine dentine, and comparable trends were obtained for enamel.
Hegazy et al., 2016 [47]	Clinical trial	Zinc–carbonate hydroxyapatite toothpaste (undeclared percentage) ZnCO_3_/n-Hap	Biorepair mouthwash can serve as a better alternative to different mouthwashes including both fluoride and chlorhexidine. This single mouthwash can serve as a multi-purpose mouthwash.
Lelli et al., 2014 [48]	In vitro/clinical trial	Zinc–carbonate hydroxyapatite toothpaste (20%) ZnCO_3_/n-HAp	In conclusion, this study demonstrates that the toothpaste containing Zn-CHA structured microcrystals, unlike nitrate potassium/sodium fluoride and non-specified fluoride toothpastes, may promote enamel superficial repair by means of the formation of a protective biomimetic CHA coating.
Butera et al., 2021 [49]	Clinical trial	Zinc–carbonate hydroxyapatite toothpaste (20%) ZnCO_3_/n-HAp	The use of toothpaste containing Zn-carbonate hydroxyapatite could be proposed as a device for domiciliary oral hygiene because the deposition of hydroxyapatite on polymeric composite resin could prevent secondary caries on the margins of restorations.
Poggio et al., 2017 [50]	In vitro	Zinc–carbonate hydroxyapatite toothpaste (20%) ZnCO_3_/n-HAp	Despite the limitations of this study, the protective pastes that showed the least weight loss due to acidic challenge are Biorepair and Regenerate.
Poggio et al., 2017 [51]	In vitro	Zinc–carbonate hydroxyapatite toothpaste (20%) ZnCO_3_/n-HAp	Toothpaste with Zn-HAP resulted in significant enamel remineralization of erosively challenged enamel, indicating that these toothpastes could provide enamel health benefits relevant to enamel erosion.
Scribante et al., 2020 [52]	In vitro	Zinc–carbonate hydroxyapatite toothpaste (30%) ZnCO_3_/n-HAp	The application of remineralizing solution induced a significant in vitro reduction of demineralized areas after the first week of application.
Butera et al., 2022 [53]	Clinical trial	Zinc–carbonate hydroxyapatite toothpaste (20%) Zinc–carbonate hydroxyapatite toothpaste (undeclared percentage)	The use of the hydroxyapatite-based toothpaste, alone or in combination with the mouthwash containing hydroxyapatite, is an effective method for the domiciliary management of dental erosion in physically active individuals such as rugby players.

### 3.2. Management of Dental Hypersensitivity [54,55,56,57,58,59,60,61,62], Table 2

Methods. The nine studies selected for the review were published in English. They are in vitro and in vivo studies conducted mainly in Italy and Germany. Other studies were conducted in China, Saudi Arabia and the United Arab Emirates.

Participants. In vitro studies used extracted human teeth, except one in vitro study that was conducted on the composition of toothpaste; in the in vivo studies, between 40 and 85 patients were selected.

Intervention. The effects of toothpaste were evaluated in the studies.

Outcomes. The use of the tested products showed efficacy in terms of occlusion of the dentinal tubules and reduction of dentinal hypersensitivity (Table 2).

**Table 2:** Studies focused on dental hypersensitivy.

Articles	Study	Agents	Conclusion
Orsini et al., 2010 [54]	Clinical trial	Zinc–carbonate hydroxyapatite toothpaste (24%) ZnCO_3_/n-HAp	This study documented that a new dentifrice containing zinc-CHA crystals significantly reduced dentinal hypersensitivity after 4 and 8 weeks, supporting its utility in clinical practice.
Orsini et al., 2013 [55]	Clinical trial	Zinc–carbonate hydroxyapatite toothpaste (30%) ZnCO_3_/n-HAp	Rapid relief from DH with a zinc–carbonate hydroxyapatite dentifrice.
Peetsch et al., 2011 [56]	In vitro	Zinc–carbonate hydroxyapatite toothpaste (undeclared percentage) ZnCO_3_/n-Hap Zinc–carbonate hydroxyapatite mouthwash (undeclared percentage) ZnCO_3_/n-HAp	The original goal to occlude the l m-sized dentinal tubules may be achievable in all cases if agglomerates are broken up under mechanical stress, e. g. during tooth-brushing. In comparison to natural tooth mineral, the Biorepair products showed the highest chemical similarity.
Steinert et al., 2020 [57]	Clinical trial	Zinc–carbonate hydroxyapatite toothpaste (20%) ZnCO_3_/n-HAp	The tested toothpaste with biomimetic HAP is well-suited for individuals suffering from dentin hypersensitivity, because subjective symptoms of dentin hypersensitivity were reduced.
Al Asmari et al., 2019 [58]	Clinical trial	Zinc–carbonate hydroxyapatite toothpaste (undeclared percentage) ZnCO_3_/n-Hap	The results suggested that the use of Zn-CHA crystals dentifrice might be an effective therapy to reduce DH.
Abou Neel et al., 2021 [59]	In vitro	Zinc–carbonate hydroxyapatite toothpaste (undeclared percentage) ZnCO_3_/n-Hap	Both ESTP (eggshell toothpaste) and TNPsESTP (titanium dioxide particle eggshell toothpaste) showed significantly higher numbers of partially occluded dentinal tubules than Biorepair.
Pei et al., 2019 [60]	In vitro	Zinc–carbonate hydroxyapatite toothpaste (undeclared percentage) ZnCO_3_/n-Hap	Hydroxyapatite-containing desensitizing toothpastes could occlude dentinal tubules effectively with a certain degree of acid resistance.
Butera et al., 2022 [61]	Clinical trial	Zinc–carbonate hydroxyapatite toothpaste (30%) ZnCO_3_/n-HAp	The hydroxyapatite-based toothpaste tested caused a reduction of hypersensitivity/pain values higher than conventional fluoride toothpaste.
Butera et al., 2022 [62]	Clinical trial	Zinc–carbonate hydroxyapatite toothpaste (24%) ZnCO_3_/n-HAp	Biomimetic zinc-hydroxyapatite showed a desensitizing effect when used to treat MIH.

### 3.3. Risk of Bias of Single Studies

Randomization, allocation concealment, blinding, outcome data, and outcome recording were evaluated, and a color was assigned according to the type of risk. A green symbol was assigned where the information was complete according to the variable considered (low risk of bias), and a yellow symbol was assigned where the information was missing or not clear (moderate risk of bias).

**Figure 2:**
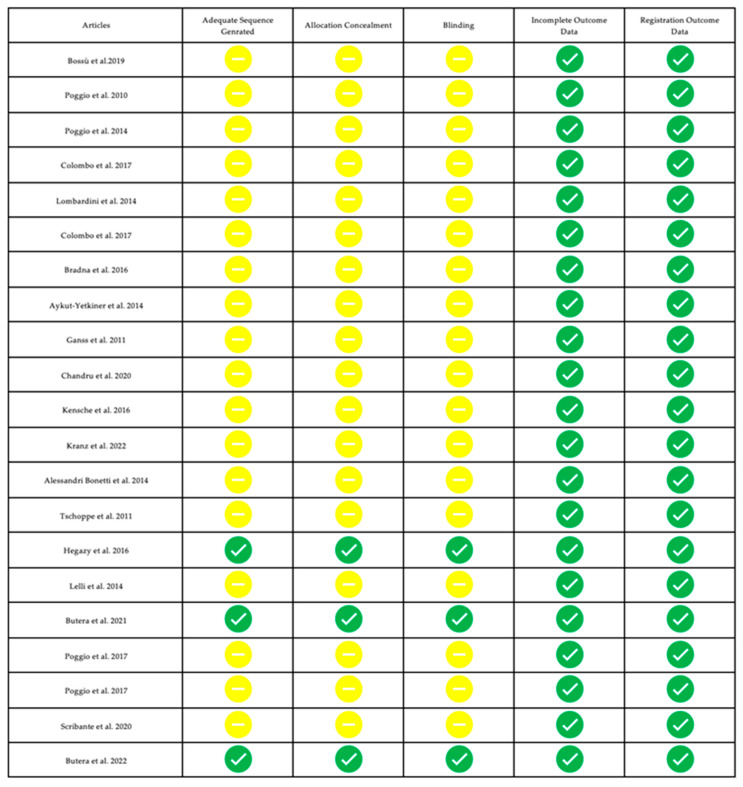
Risk of bias of studies focused on erosion and dental caries [33,34,35,36,37,38,39,40,41,42,43,44,45,46,47,48,49,50,51,52,53]. A green symbol indicates that all information was complete and the study have a low risk of bias; the yellow symbol indicates that not all information was complete (not clear or missing).

**Figure 3:**
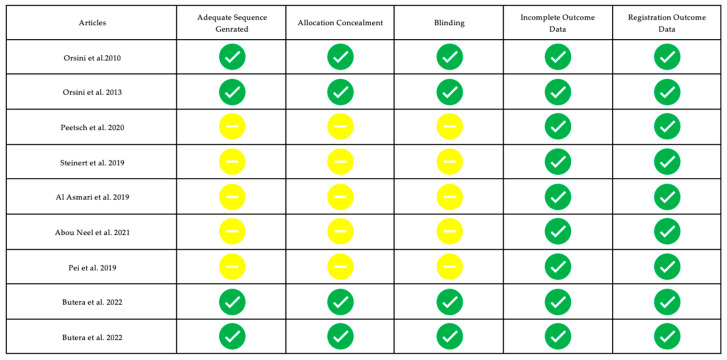
Risk of bias of studies focused on dental hypersensitivity [54,55,56,57,58,59,60,61,62]. A green symbol indicates that all information was complete and the study have a low risk of bias; the yellow symbol indicates that not all information was complete (not clear or missing).

Figure 2 and Figure 3 show the risk of bias in the main articles examined; this review presents a moderate risk of bias. Note that most of the studies were conducted in vitro.

## 4. Discussion

The biomimetic zinc–carbonate hydroxyapatite technology was developed based on the consideration that materials that mimic the natural hydroxyapatite of enamel and dentin possess good activity in terms of affinity to adhere to these biological tissues [20,21,44,63]. This hypothesis had already been explored in bone research [64].

It was hypothesized that this enhanced activity was due to a combination of factors, notably a suitable non-stoichiometric composition of zinc carbonate and hydroxyapatite, extremely reduced dimensions (microaggregate) resulting in a high surface area, a low degree of crystallinity and a specific morphology (i.e., elongated shape). The right combination of these factors was postulated to promote both increased solubility and enhanced affinity with the natural mineral composition of enamel and dentin [20,21,64].

In addition, a critical role in enhancing the reactivity of the zinc–carbonate hydroxyapatite micro-particles is attributed to the increased surface disorder on the particle surface, at which the stoichiometry of the bulk portion of the particles is no longer maintained [65].

Critical to the biological activity of this technology is the proportion of carbonate, since the carbonate ion can also be found (at a low proportion) in the structure of natural enamel and dentin hydroxyapatite. Relevant to the activity is the fact that the carbonate ion can be substituted at two different sites within the natural hydroxyapatite crystal structure: it can partially substitute for either the OH ion or the PO_4_ ion [66]. In the carbonate hydroxyapatite crystals, the carbonate substitution was designed to replace the PO_4_ ion, because this leads to a reduction of the crystallinity of the material and an increase in the solubility of the apatite phase [67].

A further advantage of the carbonate hydroxyapatite technology lies in the potential to incorporate anti-bacterial ions within the apatite structure. This is achieved by replacing a small proportion of the bivalent calcium ions in the crystal structure with, for example, zinc ions, thus arriving at a mineral complex that can be described as zinc hydroxyapatite [68]. When this material is deposited on tooth surfaces, anti-bacterial zinc ions can be released over time and exert an anti-bacterial effect on a wide range of oral pathogens [68,69].

### 4.1. Benefits for Dental Enamel

Having established the potential of zinc–carbonate hydroxyapatite to be deposited into demineralized enamel and form a new mineral layer, the interest of researchers turned to toothpaste containing zinc–carbonate hydroxyapatite for the remineralization of dental enamel and its protection from damage.

#### 4.1.1. Protection of Enamel

Several in vitro studies have highlighted the potential benefits of products such as toothpaste and mouthwashes.

Poggio et al. reported a study in which atomic force microscopy was used to investigate the erosive effect of an acidic soft drink and the repair potential of two toothpastes, one of which contained zinc–carbonate hydroxyapatite [34]. The study evaluated the effect of the two toothpastes on sound and acid-damaged enamel samples obtained from human incisors. Samples were demineralized by exposing them four times for two minutes each to a soft drink (pH 2.4); toothpaste treatment (3 min, unbrushed) was carried out four times at 0, 8, 24, and 36 h. The authors concluded that both toothpastes offered a degree of protection against erosive challenges. Similar studies were conducted showing the same result [36,38,51].

The introduction of a new technology, such as zinc–carbonate hydroxyapatite, into toothpaste invariably raises the question of how well the new products perform compared to existing technologies in the market. Lombardini et al. [37] compared the effect of four toothpastes on demineralized enamel. They noted that all technologies provided a degree of remineralization and protection against acid erosion. However, their study also raised some unexpected points; for example, one of the two toothpastes with zinc–carbonate hydroxyapatite did not perform as well as the other. A similar comparative study of the effect of different toothpaste technologies on deposition and erosion protection was reported by Bradna et al. [39]. They noted great variability in the ability of different technologies to deposit protective layers onto enamel surfaces, and high variation in protection from an acid challenge. Toothpaste with zinc–carbonate hydroxyapatite technology showed sub-microscopic deposition of minerals and limited protection against acid challenge. However, two aspects of the experimental model used by the authors are very likely to have acted against showing the full potential of the zinc–carbonate hydroxyapatite technology. Firstly, the authors used highly polished enamel as a substrate to evaluate deposition; this is not normally found in the oral environment. Secondly, the erosive challenge consisted of a single acid exposure.

#### 4.1.2. Remineralizing Effect

In vitro studies have highlighted the potential benefits of products such as toothpaste and mouthwashes.

Tschoppe et al. [46] studied the effect of different treatments, including non-fluoride toothpastes with zinc–carbonate hydroxyapatite. The results showed that treatment with toothpaste containing zinc–carbonate hydroxyapatite provided more remineralization of caries-like lesions in bovine enamel than fluoride toothpaste; the authors noted that the inclusion of zinc–carbonate hydroxyapatite in dental products might help to promote remineralization. The remineralizing effect was also evaluated in other studies [45,52].

Similar to the in vitro results are those reported by some clinical trials. Lelli et al. evaluated the effect of two toothpastes, one containing zinc–carbonate hydroxyapatite, in terms of remineralization of the enamel. The subjects included in the study followed home treatment for 8 weeks. At the end of the follow-up, some dental elements were extracted and subjected to SEM analysis. The authors confirmed that the use of a toothpaste with hydroxyapatite remineralizes the enamel by deposition of a hydroxyapatite-rich coating. Based on this property, it can be assumed that the action of zinc hydroxyapatite is also useful in protecting against carious lesions [48]. Bossù et al. focused their attention on different toothpastes to evaluate the remineralizing effect. All treatments were used 3 times a day for 15 days. Subsequently, the dental elements were extracted and evaluated at SEM; the results show that zinc–carbonate hydroxyapatite is effective in preventing carious lesions [33].

A further study addressing caries, in this case early carious lesions, was reported by Hegazy et al. [47]. The report covers a randomized clinical trial in children that compared the effectiveness of mouthwashes containing zinc–carbonate hydroxyapatite, fluoride or chlorhexidine in demineralizing early carious lesions. The results showed significant remineralization of early carious lesions in subjects using the zinc–carbonate hydroxyapatite mouthwash and in subjects using the fluoride mouthwash; therefore, the mouthwash with zinc–carbonate hydroxyapatite can serve as an alternative to mouthwashes with fluoride.

The hydroxyapatite also seems to be effective in the prevention of secondary caries on the margins of restorations [53].

### 4.2. Benefits for Dentin

It is recognized that enamel erosion has become a pre-eminent concern in recent years. The numerous studies discussed in this review have shown how a new technology can make a significant contribution to reducing and even repairing erosive damage by use of toothpaste with zinc–carbonate hydroxyapatite. Linked to the concern of enamel erosion is the area of dentin erosion, especially because increased dentin erosion can result in dentinal hypersensitivity. Recognizing the importance of this, a range of studies have been carried out to evaluate the potential of zinc–carbonate hydroxyapatite toothpaste in managing erosive damage to dentin.

A report by Poggio et al. [35] provides a clear insight into this question. It also compared the efficacy of different toothpaste technologies for the management of dentin erosion. The study aimed to evaluate the effect of toothpastes in preventing dentin erosion produced by an acidic soft drink. The results showed that all toothpaste treatments resulted in the deposition of minerals into sound and eroded dentin and tubule occlusion. There appeared to be a difference between one zinc–carbonate hydroxyapatite toothpaste and one sensitivity toothpaste compared to another zinc–carbonate hydroxyapatite toothpaste and sensitivity toothpaste. The reason for this divergence, in particular concerning the two zinc hydroxyapatite toothpastes, remained unclear. The authors concluded that all tested formulations tended to remineralize dentin surfaces.

Similar results were obtained by other studies [40,50].

Evidence from in vitro studies and clinical trials confirmed that toothpaste formulations with zinc–carbonate hydroxyapatite are able to remineralize enamel and dentin, counteract the effect of erosive damage caused by dietary acids and plaque acid and reduce dentin erosion.

### 4.3. Anti-Sensitivity Benefits

Given the strong evidence of the remineralization potential of the zinc–carbonate hydroxyapatite technology to form a new mineral layer on enamel and dentin, it became clear that this technology could also provide remineralization within dentinal tubules and thus contribute to the management of dentin hypersensitivity [56].

Occlusion of the dentinal tubules has been studied in vitro by several authors [56,59,60]; in agreement with these results, occlusion of the tubules and reduction of dentinal sensitivity are highlighted by several clinical trials. Orsini et al. [54] reported a clinical hypersensitivity study that tested this hypothesis. The aim was to evaluate the desensitizing efficacy of a new dentifrice based on zinc–carbonate hydroxyapatite. In conclusion, the authors stated that the study documented a reduction in dentinal hypersensitivity after 4 and 8 weeks due to using the dentifrice containing zinc–carbonate hydroxyapatite. Following on from the positive results from the 4-week examination within the 8-week clinical study, the authors turned their attention to the question of whether a shorter treatment time would yield similar results [55]. The aim of the study was therefore to evaluate the ability of three desensitizing dentifrices to provide rapid relief of dentinal hypersensitivity (DH). The authors concluded that the three tested dentifrices significantly reduced dentinal hypersensitivity after a 3-day treatment, supporting their use in clinical practice. Similar trials were conducted by Butera et al. [61,62].

The purpose of one of these trials was to evaluate the use of hydroxyapatite and fluoride in reducing dentinal sensitivity caused by white spots; the authors concluded that hydroxyapatite caused a reduction in hypersensitivity/pain values greater than conventional fluoride toothpaste [61]. Similar results were obtained in another MIH study [62].

Toothpaste containing zinc–carbonate hydroxyapatite can block dentin tubules and thus reduce dentin hypersensitivity.

These studies would seem to validate the purpose of this review, namely, to determine whether products containing zinc–carbonate hydroxyapatite can be considered a viable alternative to fluorine or other prophylactic systems in terms of remineralization. It is known based on the literature that fluoride is a remineralizing agent [27,28,29]. CCP-ACP also appears to be another valuable system [30,31,32,70] for reducing dental sensitivity [71]. Several in vitro studies included in this review have characterized most of the findings from the database research.

In the future, it would be useful to develop more clinical studies in order to confirm the results obtained with greater certainty.

## 5. Conclusions

Most of the studies included in this review confirmed that toothpaste with biomimetic zinc–carbonate hydroxyapatite is effective in remineralizing dental enamel by deposition of a hydroxyapatite-rich coating, and in reducing dentin erosion by the deposition of minerals onto sound and eroded dentin. Furthermore, it was demonstrated that toothpaste containing zinc–carbonate hydroxyapatite can block dentin tubules and thus reduce dentin hypersensitivity in a shorter treatment time.

## Data Availability

The data presented in this study are available upon request to the corresponding author.
